# Determinants of immunization inequality among urban poor children: evidence from Nairobi’s informal settlements

**DOI:** 10.1186/s12939-015-0154-2

**Published:** 2015-02-27

**Authors:** Thaddaeus Egondi, Maharouf Oyolola, Martin Kavao Mutua, Patricia Elung’ata

**Affiliations:** African Population and Health Research Center (APHRC), Nairobi, Kenya; Department of Public Health and Clinical Medicine, Epidemiology and Global Health, Umeå University, Umeå, Sweden

**Keywords:** Immunization, Health inequality, Concentration index, Urban poor, Kenya

## Abstract

**Introduction:**

Despite the relentless efforts to reduce infant and child mortality with the introduction of the National Expanded Programmes on Immunization (EPI) in 1974, major disparities still exist in immunizations coverage across different population sub-groups. In Kenya, for instance, while the proportion of fully immunized children increased from 57% in 2003 to 77% in 2008–9 at national level and 73% in Nairobi, only 58% of children living in informal settlement areas are fully immunized. The study aims to determine the degree and determinants of immunization inequality among the urban poor of Nairobi.

**Method:**

We used data from the Nairobi Cross-Sectional Slum Survey of 2012 and the health outcome was full immunization status among children aged 12–23 months. The wealth index was used as a measure of social economic position for inequality analysis. The potential determinants considered included sex of the child and mother’s education, their occupation, age at birth of the child, and marital status. The concentration index (CI) was used to quantify the degree of inequality and decomposition approach to assess determinants of inequality in immunization.

**Results:**

The CI for not fully immunized was −0.08 indicating that immunization inequality is mainly concentrated among children from poor families. Decomposition of the results suggests that 78% of this inequality is largely explained by the mother’s level of education.

**Conclusion:**

There exists immunization inequality among urban poor children in Nairobi and efforts to reduce this inequality should aim at targeting mothers with low level of education during immunization campaigns.

## Introduction

The Expanded Programme on Immunization was launched in 1974 with the overarching goal of reducing infant mortality around the world and more specifically in developing countries. Today, significant progress has been made and the proportion of fully immunized children (FIC) has reached 83% [1,2]. Despite these efforts, disparities in FIC coverage across countries and subpopulations still exist. In sub-Saharan Africa, for instance, while some countries have reached FIC coverage of 99%, others are lagging with coverage below 50%. Furthermore, studies have highlighted inequalities in FIC coverage among households as well as between rural and urban areas [3,4] in countries with high coverage. Whereas immunization coverage has increased overall in many countries, children living in rural and urban informal settlements areas have recorded the lowest immunization coverage. Various explanations have been put forward about immunization inequality among subpopulations. Some studies have underscored the importance of mother’s education and socioeconomic status of the household [5,6], indirect costs associated with immunization such as transportation and opportunity costs [7,8] in explaining immunization inequality.

To realize the effort towards FIC coverage, there is need to identify determinants of the existing disparities. In Kenya, FIC coverage are higher in urban than rural areas (81% and 76% respectively) [9]. While FIC coverage in Nairobi is at 73%, children in informal settlement of the same city have a coverage of 58% [10]. Moreover, children in informal settlement areas are the most vulnerable and exposed to major health risks [11,12]. These areas are characterized by abject poverty and precarious living conditions [13] which predispose residents to higher risk of morbidity and mortality. Therefore, increasing immunization coverage is tantamount to increasing child survival among the urban poor who have poor access health care services. Moreover, assessing inequality among the urban poor is crucial since informal settlement dwellers are not homogenous groups but differ in access to services. Therefore, understanding immunization inequality among the urban poor is necessary in identifying appropriate interventions for urban poor which is about 60% of the urban population.

Wealth index has been used to capture the socio-economic status of household [14,15] while concentration and human opportunity indices have been used in explaining inequalities across and within countries [4,16]. The concentration index (CI) has the advantage of not only providing a measure of inequality but also allows us to identify the contributing factors to the inequality. Since the objective of this study is to identify the contributing factors to immunization inequality, CI was used.

The objective of this study is to tease out factors that might potentially explain the inequality in FIC coverage observed in the urban informal settlements of Nairobi. Previous studies in Kenya focused either on single vaccine [17] or the general health inequality [12]. This paper contributes to the existing literature by not only concentrating on one of the most vulnerable segment of the Kenyan population but also in understanding immunization disparity among the urban poor. Secondly, the study helps us understand factors associated with the high immunization inequality within the urban poor population. Overall, the paper provides the groundwork for policymakers to develop policy responses that target population segments with lower immunization coverage in the effort to achieve universal coverage.

## Methods

### Data

The paper takes advantage of the second Nairobi Cross-Sectional Slum Survey (NCSS II) data collected by the African Population and Health Research Center (APHRC) from all informal settlements of Nairobi in 2012. The NCSS II was conducted in 2012 in all informal settlements in Nairobi. A total of 3892 women aged 15–49 years were interviewed from 5490 households during the survey. In addition to socio-demographic characteristics of the households and women, information on vaccination was collected from all women who had a living child born during the last five years preceding the survey. We use data on 382 children aged 12–23 months who were expected to have received all the recommended vaccinations. Information on vaccination status of the child was obtained from either vaccination cards or by interviewing the mother or caregiver where a card was not available.

FIC was defined as a child aged between 12 and 23 months who received all the routine childhood vaccinations as recommended by World Health Organization (WHO). The detailed description on how the information status was obtained and the assumptions that were made is given elsewhere [18]. To capture the socio-economic status of the households, an assets index was constructed using the principal component analysis (PCA) based on different household assets and amenities. The generated wealth score was grouped into tertiles as a measure of socio-economic status with the first tertile representing the poorest group and the last tertile representing the least poor group. The predictor variables were: sex and birth order of the child, mother’s education, involvement in income generating activity (IGA), marital status, age at birth of the child and ethnic group.

### Inequality and decomposition analysis

Previous studies have estimated immunization inequality across wealth index strata using CIs [19,20]. The general CI is revised for binary health outcomes [21]. The detailed description of the formula and related components are given elsewhere [19-21]. A positive CI implies that the health variable is more concentrated among the better-off population while a negative value indicates pro-poor inequality. The decomposition of CI was carried out using a regression-based approach [22] and then revised for the binary outcome [21]. All computations were done using STATA version 12.1 and were adjusted for sampling weights. The standard errors and corresponding confidence intervals of the estimates were computed using bootstrap approach.

## Results

There were slightly more male children compared to females (52% vs.48%). 43% of children were first born with only 10% of children being either birth order 4 or 5. Half of the mothers had at least secondary education and 19% had either no or incomplete primary. There were more mothers from Luhya ethnic group compared to other ethnic groups and most mothers (85%) were married. About a half of the mothers were involved in IGA and only about 6% engaged in formal employment. There were more young mothers aged less 25 years at time of the child’s birth (57%) than old mothers.

Table 1 shows that the proportion of children not FIC seemed to reduce by higher birth order and same was observed with the level of education. The proportion not FIC did not differ by either ethnic groups or mother’s marital status. The proportion of not FIC children was high among young mothers (32% vs 28%) and was also higher in the lower social class compared to higher classes.

**Table 1:** **Summary distribution of children and proportion not fully immunized by determinants**

	**% of children**	**% not fully immunized**	**no. of children**
Sex of the child			
Female	48.2	34.8	184
Male	51.8	26.8	198
Child's birth order			
1	42.8	32.5	163
2-3'	47.2	30.6	180
4-5'	10.0	23.7	38
Mother's level of education			
No/primary incomplete	18.6	29.6	71
Primary level	31.5	35.8	120
Secondary plus	49.9	27.9	190
Ethnic group			
Kamba	20.9	31.3	80
Kikuyu	19.9	32.9	76
Luhya	27.2	31.7	104
Luo	16.8	28.1	64
Other	15.2	27.6	58
Main IGA of mother			
Business	19.6	32.0	75
Informal	18.6	26.8	71
Formal	5.8	22.7	22
Unemployed	49.7	32.6	190
Missing	6.3	29.2	24
Whether in union or not			
Married	85.1	30.5	325
Never married	14.9	31.6	57
Mother’s age at birth of the child			
<20	17.0	32.3	65
20-24	40.1	32.0	153
25-34	38.2	28.8	146
35+	4.7	27.8	18
Wealth index			
Poorest	27.4	36.9	103
Poor	29.8	31.3	112
Least poor	42.8	26.1	161

**Figure 1:**
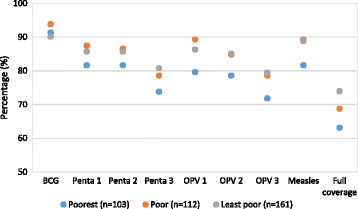
**The level of coverage for individual vaccines by the wealth status.**

Figure 1 shows large disparity for Pentavalent, Oral polio vaccine (OPV) and Measles with poorest households having low values for all the vaccines. The overall coverage among the poorest household (63%) was lower compared to the poor (69%) and the least poor (74%) households. The coverage for each antigen separately were consistently lower in poorest households as compared to the poor and the least poor households.

Table 2 presents CIs of the possible determinants and their percentage contributions to immunization inequality. The CIs for both dependent and determinants provide insights on immunization inequality. The CI value for child not fully immunized is −0.08 among the urban poor which indicates that immunization practice is less among children from the poorest families. The observed immunization inequality among the urban poor was statistically significant (95% CI −0.083:-0.077). Among the determinants considered, it was observed that birth order one, mothers with low level of education, mothers not involved in any IGA, mothers giving birth at young age concentrates more among the poorest families. Mother’s level of education was a major contributor to overall inequality by 78%. The other important contributors were birth order (18%) and involvement in any IGA (22%). The result also indicates that the degree of health inequality in terms of child immunization is less determined by marital status or ethnic groups.

**Table 2:** **Concentration indices and contributions of determinants to immunization inequality**

	**Concentration index**			**Percentage contribution**		
	**Estimate**	**95% CI**		**Estimate**	**95% CI**	
Not fully immunized	−0.080	−0.083	−0.077			
Sex (*male*)	0.007	0.005	0.009	14%	5%	23%
Birth order (*order 1*)	−0.028	−0.030	−0.026	18%	12%	24%
Mother's education level (*primary or less*)	−0.080	−0.082	−0.078	78%	58%	98%
Mother's employment status (*not involved in IGA*)	−0.057	−0.058	−0.055	22%	12%	31%
Marital status (*not in union*)	0.021	0.017	0.026	−6%	−10%	−2%
Mother age at birth of the child (*<25 years*)	−0.035	−0.037	−0.033	13%	4%	22%
Ethnic group (*ref: other*)						
Kamba	−0.085	−0.089	−0.082	−16%	−27%	−5%
Kikuyu	0.165	0.162	0.169	−34%	−53%	−15%
Luhya	−0.041	−0.045	−0.038	11%	−2%	24%
Luo	0.004	0.000	0.009	8%	4%	13%

## Discussion

This paper shed lights on some determinants of immunization inequality in the informal settlements of Nairobi. The study shows a third of children were not fully immunized. Large disparity in individual vaccines was observed for measles, Pentavalent and OPV vaccines. The CI results indicate that more children are not fully immunized from the poorest households. Among the determinants considered; birth order, level of education, IGA and age correlates with the poorest. The study also identified mother’s level of education as major contributor to immunization inequalities.

The study provides evidence on the decomposition of socioeconomic inequality in child immunization among the urban poor population in Nairobi. Data suggest that coverage rates among urban poor children largely living in informal settlements are lower than the overall urban average [23]. This is in resonance with other studies done in similar setting showing that the poorest were more likely not to complete the recommended vaccinations [9,10]. The study findings show that slum populations cannot be considered homogenous. The finding of mother’s level of education as major contributor is consistent with previous studies carried out in India and Bangladesh [16,24]. Given that large proportions of mothers in this setting have low level of educational attainment, it is imperious to find other means of sensitizing this already vulnerable segment of the Kenyan population.

In Kenya, the routine childhood immunizations are free, which should minimize inequality related to wealth. However, our analysis suggests that wealth still remains an important factor in access to full vaccination even among the poor population. There are several possible explanations for this observation among poor populations. One possible explanation is that poor people may prefer to spend time on income earning opportunities rather than on accessing preventive health services, such as immunization whose long-term preventive benefits are less tangible [25]. Secondly, health seeking attitudes and practices of poor households may also explain the inequalities. Thirdly, indirect costs, such as those accrued for travel to immunization centers or time lost from IGAs may hinder seeking of vaccination services. Disparities exist across ethnic groups with immunization inequality concentrated among Luhyas and Luos. This disparity might be explained by the differences in such determinants as education and income levels alongside cultural differences.

Overall, the study underscores the important role of the mother in the immunization of their children. The results have implications that policy makers need to be aware of existence of disparities even among urban poor. The results supports the idea that health intervention strategies aiming at reducing socioeconomic immunization inequality could benefit from being supplemented with strategies aimed at poverty and illiteracy reduction. Moreover, community perception and attitude analysis is required to understand deeper the barriers in full immunization among sub-populations with low coverage.
